# Rates of diabetic retinopathy among cluster analysis—identified type 2 diabetic mellitus subgroups

**DOI:** 10.1007/s00417-023-06260-5

**Published:** 2023-10-16

**Authors:** Rachel A. Scott, Vivian I. Lu, Nathan Grove, Jennifer L. Patnaik, Niranjan Manoharan

**Affiliations:** 1 Department of Ophthalmology, University of Colorado School of Medicine, 1675 Aurora Court, Aurora, CO 80034 USA; 2 University of Colorado School of Medicine, Aurora, CO USA

**Keywords:** Diabetic retinopathy, Retina, Type 2 diabetes mellitus, Complications of diabetes, Vitrectomy, Panretinal photocoagulation

## Abstract

**Purpose:**

To determine whether phenotypic clustering of patients with diabetes mellitus (DM) is associated with more advanced diabetic retinopathy (DR).

**Methods:**

Retrospective cohort study of 495 patients with no prior DR treatment seen at a tertiary care clinic 2014–2020. Four previously identified clusters from Ahlqvist’s 2018 paper were reproduced utilizing baseline hemoglobin A1c, body mass index, and age at DM diagnosis. Age-adjusted Cox proportional hazard ratios were used to compare clusters with reference as the lowest risk cluster.

**Results:**

All four type 2 DM clusters were replicated with our cohort. There was a significant difference in racial distribution among clusters (*p* = 0.018) with severe insulin-resistant diabetes (SIRD) having the higher percentage of Caucasians and lower percentage of Hispanics compared to other groups and a higher percentage of African Americans comprising the severe insulin-deficient diabetes (SIDD) cluster than other groups. Rates of proliferative diabetic retinopathy were higher in mild obesity-related diabetes (MOD) (28%), SIDD (24%), mild age-related diabetes (MARD) (20%), and lowest in SIRD (7.9%), overall *p* = 0.004. Rates of vitreous hemorrhage were higher in MOD (*p* = 0.032) and MARD (0.005) compared to SIRD.

**Conclusion:**

Baseline clinical measures may be useful in risk stratifying patients for progression to retinopathy requiring intervention.


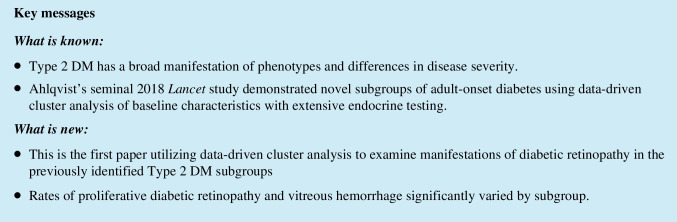


## Introduction

As more is understood about the underlying pathophysiology of diabetes mellitus (DM), it has become clear that type 1 and type 2 are not sufficient in distinguishing the different phenotypes that present due to the complexity of the disease. An emerging body of literature in endocrinology has set out to define the nuances within the catch-all diagnosis that is type 2 diabetes mellitus (T2DM).

Ahlqvist’s seminal 2018 *Lancet* study, utilized 8980 newly diagnosed diabetic patients from the Swedish All New Diabetics in Scania (ANDIS) cohort followed over 10 years, demonstrated novel subgroups of adult-onset diabetes using data-driven cluster analysis of baseline characteristics [1]. The six variables of age at diagnosis, body mass index (BMI), hemoglobin A1c (HbA1c), glutamate decarboxylase antibodies, and homeostatic model assessment 2 estimates of beta-cell function and insulin resistance (HOMA-2B, HOMA-2IR) were used to create five clusters. These include severe autoimmune diabetes (SAID), severe insulin deficient diabetes (SIDD), severe insulin resistant diabetes (SIRD), mild obesity-related diabetes (MOD), and mild age-related diabetes (MARD).

SAID was defined by the presence of glutamic acid decarboxylase antibodies (GADA) as present in type 1 diabetes and latent autoimmune diabetes as well as characterized by low insulin secretion and poor metabolic control. SIDD, while lacking auto-antibodies, was also identified by low insulin secretion and poor metabolic control with the associated findings of increased risk of the microvascular processes of retinopathy and neuropathy. Interestingly, SIRD—defined by its insulin resistance, late onset, and association with obesity—was associated with nephropathy, another microvascular condition, as well as fatty liver disease. MOD was associated with obesity and early age of onset, while MARD was defined by its late age of onset, mild metabolic derangements, and low risk of complications [1].

These clusters have since been reproduced in several studies which have included more diverse populations [2DDI. Of note, Kahkoska and colleagues were able to reproduce the clusters retrospectively from three global cardiovascular outcomes trials using just three readily attainable variables: age at diagnosis, BMI, and HbA1c [2]. These cited studies assessed numerous outcome variables including risk of diabetic complications. However, one shared limitation of assessment of diabetic complications was treating diabetic retinopathy (DR) as a binary outcome: present or absent. The severity of and complications related to DR are on a spectrum, secondary to the degree of capillary leakage, capillary occlusion, and retinal ischemia that underlie the disease process [3]. Retinal ischemia in DR ultimately leads to sequelae including retinal neovascularization, vitreous hemorrhage (VH), tractional retinal detachment (TRD), and neovascular glaucoma (NVG).

This project sought to assess whether clusters were reproducible in a retrospective cohort of patients with DR and if these clusters were associated with risk of advanced DR.

## Methods

This retrospective cohort study was approved by the Colorado Multiple Institutional Review Board and the study complied with the Health Insurance Portability and Accountability Act of 1996 and the Declaration of Helsinki. Patients seen at the Denver Health Eye Clinic at Denver Health Medical Center (DHMC) (Denver, Colorado) between January 2014 and December 2020 with any stage of DR were considered eligible for inclusion. Inclusion criteria were a complete electronic medical record (EMR) and DR treatment-naïve status. Data was captured for initial entry into the county hospital ophthalmology clinic as well as annual follow-up points as available.

Exclusion criteria were deceased status at time of data collection, type 1 DM diagnosis, other significant ocular pathology, correctional facility status, and/or absence of the three required cluster variables: HbA1c, body mass index (BMI), and age at DM diagnosis. Because type 1 DM diagnosis was an exclusion criteria and GADA antibodies were not available, the SAID cluster was not identified or analyzed in this study.

For each patient who met inclusion criteria, the following variables were abstracted into a secure database: age, age at time of diabetes diagnosis, sex, race/ethnicity, diagnosis of hypertension, diagnosis of chronic kidney disease, HbA1c, BMI, microalbumin, albumin: creatinine ratio, lipid panel, alanine transaminase (ALT), DR severity at initial and final visits, NVG, VH, diabetic macular edema (DME), and DR treatment received. Both eyes of each patient were included in the study. When an event occurred in one eye, the patient remained in the study and contributed data from the fellow eye.

Following Kahkoska and colleagues’ method in validation of T2DM clusters in the DEVOTE, LEADER, and SUSTAIN cardiovascular outcomes trials [2], we utilized the techniques described below. Scaled and centered values of the baseline HbA1c, baseline BMI, and age at the time of T2DM diagnosis were used for clustering [2]. Males and females were assigned to clusters separately based on the shortest Euclidean distance from values of each individual’s clustering variables to cluster centroids previously identified by Ahlqvist, who used a K-means clustering algorithm to find centroids [1]. Cluster performance was assessed by computing the subject-specific ratio of the Euclidean distance to the assigned cluster centroid to the Euclidean distance to the next closest cluster centroid. A ratio closer to 0 indicates a strong association between a subject and the assigned cluster, and a ratio closer to 1 indicates a weak association [2].

Basic frequencies and percentages are presented for categorical variables. Means, standard deviations (SD), and medians are presented for continuous variables. The summary measures are presented for the entire cohort and for each cluster group. Demographic and clinical data were analyzed for differences across clusters using the Kruskal-Wallace rank sum test for continuous variables, and Fisher’s exact test for categorical variables. Post-hoc pairwise comparisons to assess differences in rates of proliferative diabetic retinopathy (PDR) and VH between clusters were performed using Fisher’s exact test with Bonferroni-corrected *p*-values. To determine the association between PDR and variables of interest, unadjusted logistic regressions and logistic regressions adjusted for assigned cluster were fitted. Differences in baseline HbA1c and the mean of 1–3 years of follow-up HbA1c were assessed using Welch’s two-sample *t*-test. Statistical analysis was performed using R version 4.1.3 [4]. *p*-values less than 0.05 indicated statistical significance for this study.

## Results

**Fig. 1:**
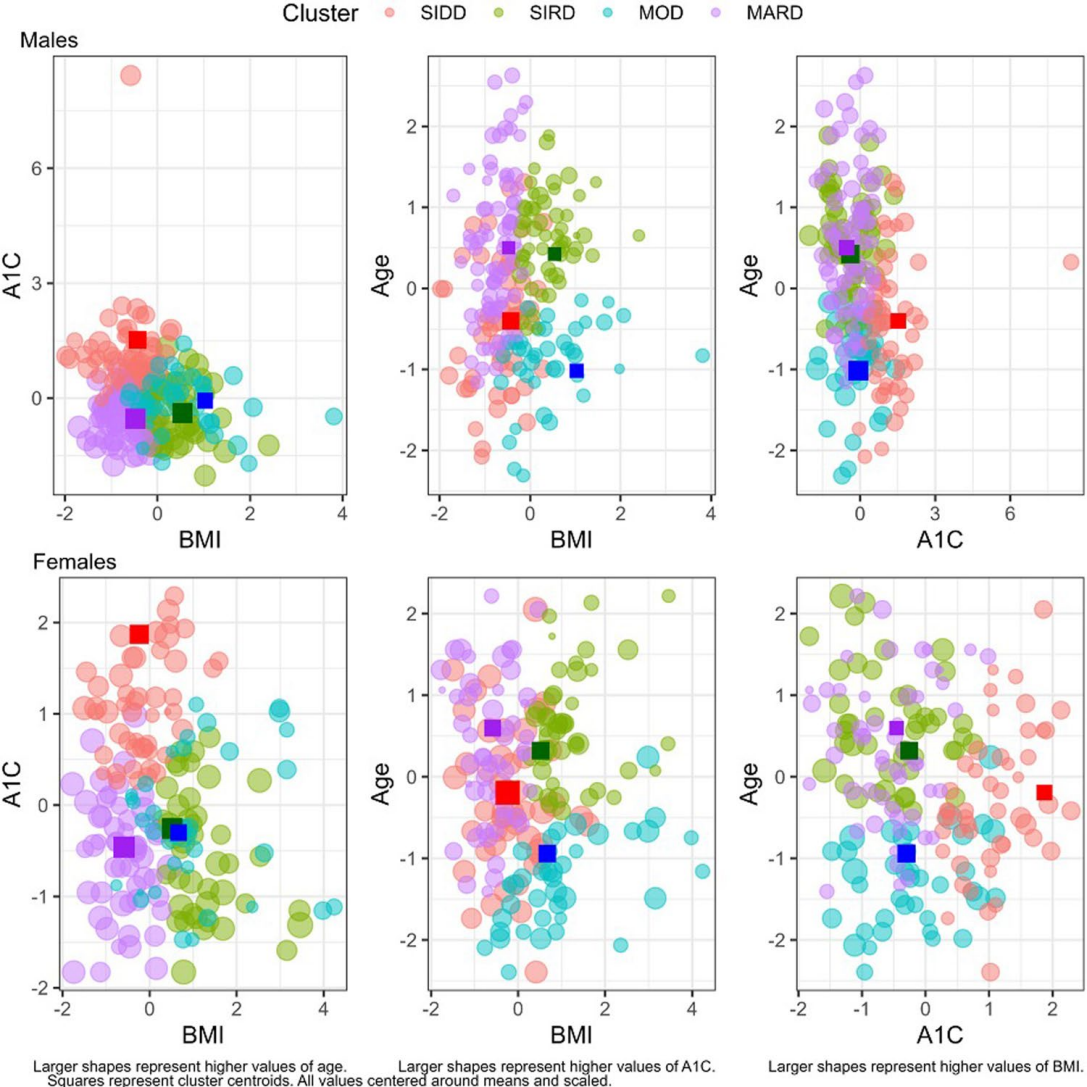
Cluster plot. Visualization of clustering by combinations of clustering variables. Subjects were assigned clusters based on the smallest Euclidean distance from a subject to the nearest cluster centroid. Clustering variables include age at DM diagnosis, baseline BMI, and baseline A1C. Values for each variable were centered around their mean and scaled to a standard deviation of 1. Squares in each panel represent cluster centroids identified by Ahlqvist, 2018. Sizes of shapes in each panel represent larger values of the clustering variable not included in the axes of the panel

**Fig. 2:**
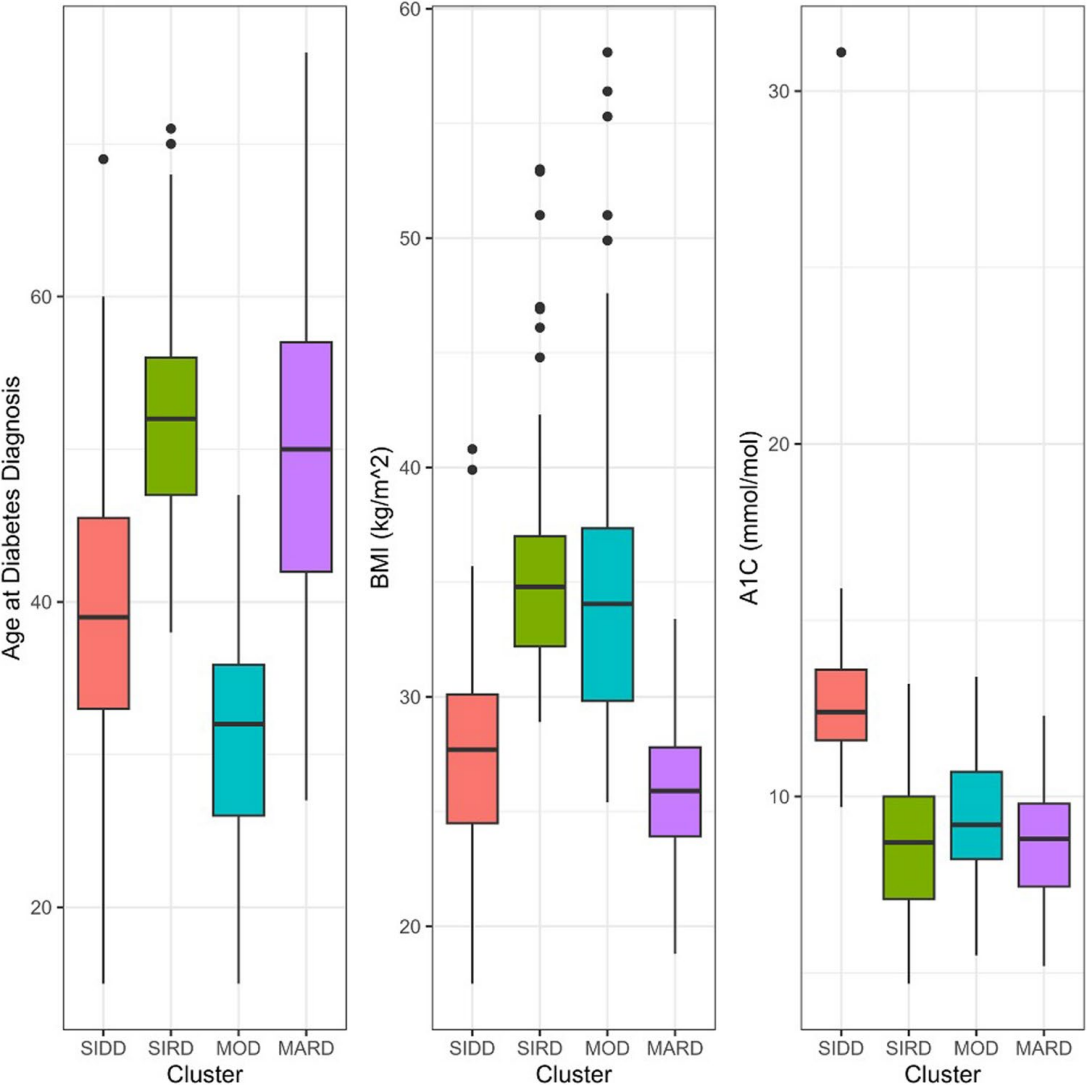
Box plot. Box plots showing distribution of clustering variables among clusters. Boxes represent the 25th, median, and 75th percentiles. Whiskers extend 1.5 times the interquartile range

Four hundred sixteen DR treatment-naïve patients were seen for DR between January 2014 and December 2020 and met inclusion criteria. One hundred three subjects were assigned to SIDD, 101 subjects were assigned to SIRD, 78 subjects were assigned to MOD, and 134 subjects were assigned to MARD. Figure 1 provides a visualization of clustering by combinations of clustering variables. The mean and median of the distance ratio of assigned cluster centroid to next closest cluster centroid (described in more detail above) was 0.685 (SD = 0.205), and 0.710 (IQR = 0.306). The distribution of the three clustering variables among the four clusters is further illustrated by box plot of Fig. 2.

Baseline characteristics stratified by cluster are presented in Table 1. While 55% of the cohort was male, gender did not significantly vary between cohorts (*p* = 0.590). The mean age of study patients at study enrollment was 57.6 (SD = 10.2) years and varied significantly across clusters in an all group comparison (*p* < 0.001) with the MOD cluster having the lowest average age at 49.7 (SD = 8.9) years. There was a high percentage of patients reporting Hispanic ethnicity (63%) in our study cohort. Race/ethnicity also significantly varied across clusters (*p* = 0.012).

**Table 1:** Demographic and clinical factors

Variable	Overall *N* = 416	SIDD *N* = 103	SIRD *N* = 101	MOD *N* = 78	MARD *N* = 134	*p*-value
Age at enrollment (years)						**< 0.001**^**1**^
Mean (SD)	57.6 (10.2)	53.6 (9.5)	62.0 (7.3)	49.7 (8.9)	62.0 (9.2)	
Median (IQR)	58.0 (15.0)	53.0 (13.0)	63.0 (10.0)	50.5 (11.5)	64.0 (13.0)	
Sex, *n* (%)						0.565^2^
Male	229 (55%)	54 (52%)	55 (54%)	40 (51%)	80 (60%)	
Female	187 (45%)	49 (48%)	46 (46%)	38 (49%)	54 (40%)	
Race/ethnicity, *n* (%)						**0.018**^**2**^
White	64 (15%)	13 (13%)	24 (24%)	9 (12%)	18 (13%)	
Black or African American	55 (13%)	20 (19%)	14 (14%)	6 (7.7%)	15 (11%)	
Hispanic or Latino	261 (63%)	65 (63%)	56 (55%)	56 (72%)	84 (63%)	
Asian	22 (5.3%)	3 (2.9%)	3 (3.0%)	2 (2.6%)	14 (10%)	
Other	14 (3.4%)	2 (1.9%)	4 (4.0%)	5 (6.4%)	3 (2.2%)	
Hypertension, *n* (%)	322 (79%)	74 (73%)	84 (84%)	55 (72%)	109 (83%)	0.072^2^
Unknown	8	2	1	2	3	
Chronic kidney disease, *n* (%)						**0.034**^**2**^
Yes	153 (37%)	49 (48%)	29 (29%)	28 (36%)	47 (35%)	
No	236 (57%)	49 (48%)	69 (68%)	42 (54%)	76 (57%)	
Uncertain	27 (6.5%)	5 (4.9%)	3 (3.0%)	8 (10%)	11 (8.2%)	
Baseline HbA1c (mmol/mol)						**< 0.001**^**1**^
Mean (SD)	9.8 (2.5)	12.8 (2.3)	8.7 (1.8)	9.4 (1.8)	8.6 (1.5)	
Median (IQR)	9.6 (3.2)	12.4 (2.0)	8.7 (2.9)	9.2 (2.5)	8.8 (2.3)	
Baseline BMI (kg/m^2)						**< 0.001**^**1**^
Mean (SD)	30.4 (6.5)	27.5 (4.4)	35.4 (4.9)	35.4 (7.4)	25.9 (2.9)	
Median (IQR)	29.2 (8.1)	27.7 (5.6)	34.8 (4.8)	34.0 (7.5)	25.9 (3.9)	
Age at DM diagnosis (years)						**< 0.001**^**1**^
Mean (SD)	44.1 (12.1)	39.4 (10.1)	51.7 (7.3)	30.8 (6.9)	49.6 (11.0)	
Median (IQR)	44.0 (17.8)	39.0 (12.5)	52.0 (9.0)	32.0 (9.9)	50.0 (15.0)	
Baseline microalbumin (mg/dL)						0.074^1^
Mean (SD)	48.3 (127.6)	46.0 (102.1)	23.7 (71.6)	71.3 (134.5)	57.5 (169.8)	
Median (IQR)	4.7 (27.0)	6.0 (32.8)	3.0 (9.1)	6.0 (49.9)	5.2 (27.2)	
Unknown	76	17	13	18	28	
Albumin creatinine ratio						**0.019**^**1**^
Mean (SD)	666.4 (1,661.6)	847.9 (1,858.1)	219.7 (506.5)	874.7 (1,726.1)	765.5 (1,999.4)	
Median (IQR)	65.2 (388.0)	146.1 (591.4)	39.4 (144.1)	58.5 (780.7)	64.4 (389.8)	
Unknown	100	22	20	21	37	
Cholesterol (mg/dL)						**0.006**^**1**^
Mean (SD)	185.9 (83.0)	198.4 (54.3)	173.1 (50.8)	203.0 (155.0)	175.5 (52.3)	
Median (IQR)	177.0 (70.0)	189.0 (63.0)	169.5 (69.8)	176.0 (62.0)	170.0 (79.5)	
Unknown	31	6	5	5	15	
Triglycerides (mg/dL)						0.116^1^
Mean (SD)	235.7 (270.7)	242.5 (184.1)	198.2 (116.6)	302.8 (528.5)	219.4 (159.0)	
Median (IQR)	178.0 (150.0)	196.0 (164.0)	160.5 (108.5)	197.0 (162.0)	157.0 (142.5)	
Unknown	31	6	5	5	15	
HDL (mmol/L)						0.163^1^
Mean (SD)	44.0 (12.7)	45.2 (12.6)	44.2 (12.9)	40.7 (11.9)	44.7 (12.9)	
Median (IQR)	42.0 (13.0)	44.0 (13.0)	42.0 (13.5)	40.0 (17.0)	42.0 (14.5)	
Unknown	33	6	5	7	15	
LDL (mmol/L)						**0.009**^**1**^
Mean (SD)	95.1 (40.4)	106.9 (36.1)	89.4 (39.9)	88.9 (38.1)	94.4 (43.9)	
Median (IQR)	93.0 (53.0)	103.0 (48.0)	84.5 (49.5)	86.0 (51.5)	95.0 (62.5)	
Unknown	72	20	9	15	28	
HDL/LDL ratio						0.122^1^
Mean (SD)	0.6 (0.4)	0.5 (0.2)	0.6 (0.5)	0.6 (0.3)	0.6 (0.4)	
Median (IQR)	0.5 (0.3)	0.4 (0.2)	0.5 (0.4)	0.5 (0.4)	0.5 (0.3)	
Unknown	73	20	9	16	28	
ALT (IU/L)						**0.043**^**1**^
Mean (SD)	38.3 (49.6)	30.2 (29.6)	43.8 (63.7)	37.3 (31.9)	41.2 (57.4)	
Median (IQR)	28.0 (17.0)	25.0 (11.0)	30.0 (24.0)	28.5 (17.2)	28.0 (17.5)	
Unknown	25	2	4	8	11	

Bold highlight statistical significance *p* <0.05

^1^Kruskal-Wallis rank sum test

^2^Fisher’s exact test for count data with simulated *p*-value (based on 2000 replicates)

Abbreviations for the clusters above refer to *SIDD* severe insulin deficient diabetes, *SIRD* severe insulin resistant diabetes, *MOD* mild obesity-related diabetes, and *MARD* mild age-related diabetes

Albumin:creatinine ratio (ACR) was significantly different across cohorts (*p* = 0.019). SIRD had the lowest ACR, 219.7 (SD = 506.5) and also had the lowest proportion of diagnosis of chronic kidney disease at 29%, which differed across cohorts (*p* = 0.033). LDL was significantly different across clusters (*p* = 0.009) and was highest in SIDD with an average of 106.9 (SD = 36.1) mmol/L. ALT was also significantly different across clusters (*p* = 0.043) and was highest in SIRD at 43.8 (SD = 63.7) IU/L. SIDD had the greatest reduction in HbA1c (*p* < 0.001) when comparing HbA1c at time of establishing care at the county hospital to the average over the following 3 years (shown in Table 3). This corresponds to SIDD having the highest A1c at presentation.

The incidence of stages of DR and its sequelae including VH, DME, and NVG is shown by the cluster in Table 2. A significant difference across clusters was found for severity of DR (*p* = 0.004) and incidence of VH (*p* = 0.007). Post hoc pairwise comparisons of the proportion of PDR by cluster showed that SIDD (24%) and MOD (28%) had significantly higher rates of PDR compared to the cluster with the lowest rate of 7.9%, SIRD (Bonferroni adjusted *p*-values 0.012 and 0.003, respectively) (Table 4). When adjusted for cluster assignment, age at DM diagnosis (OR = 0.969, 95% CI: 0.942–0.995), baseline microalbumin (OR = 1.003, 95% CI: 1.001–1.006), and ACR (OR = 1.0003, 95% CI: 1.0001–1.0004) were significantly associated with the odds of PDR (*p* = 0.022, *p* = 0.002, *p* < 0.001, respectively) (Table 5). Increasing age at DM diagnosis was associated with reduction in the odds of PDR. Higher levels of baseline microalbumin and higher albumin-creatinine ratio were associated with increase in the odds of PDR. Table 6 shows post hoc pairwise comparisons of the proportion of VH by cluster in which MOD and MARD had significantly higher proportions of VH compared to SIRD (Bonferroni adjusted *p*-values 0.032 and 0.005). Rates of DME did not vary across clusters (Table 2).

**Table 2:** Ocular characteristics

Variable	Overall *N* = 416	SIDD *N* = 103	SIRD *N* = 101	MOD *N* = 78	MARD *N* = 134	*p*-value
Diabetic retinopathy, *n* (%)						**0.006**^**1**^
No diabetic retinopathy	58 (14%)	10 (9.7%)	17 (17%)	14 (18%)	17 (13%)	
Mild NPDR	155 (37%)	29 (28%)	50 (50%)	22 (28%)	54 (40%)	
Moderate NPDR	90 (22%)	29 (28%)	21 (21%)	13 (17%)	27 (20%)	
Severe NPDR	31 (7.5%)	10 (9.7%)	5 (5.0%)	7 (9.0%)	9 (6.7%)	
PDR	82 (20%)	25 (24%)	8 (7.9%)	22 (28%)	27 (20%)	
Neovascular glaucoma, *n* (%)	4 (1.0%)	2 (1.9%)	1 (1.0%)	1 (1.3%)	0 (0%)	0.407^1^
Vitreous hemorrhage, *n* (%)	41 (9.9%)	10 (9.7%)	2 (2.0%)	10 (13%)	19 (14%)	**0.005**^**1**^
Diabetic macular edema, *n* (%)	53 (13%)	14 (14%)	8 (7.9%)	11 (14%)	20 (15%)	0.368^1^

Bold highlight statistical significance *p* <0.05

^1^Fisher’s exact test for count data with simulated p-value (based on 2000 replicates)

**Table 3:** Patient HbA1c measures at baseline and 1–3-year follow-up by cluster

Cluster	Mean baseline HbA1c	Mean 1–3 year HbA1c	*p*-value
Total	9.8	8.8	< 0.001
SIDD	12.8	9.7	< 0.001
SIRD	8.7	8.4	0.262
MOD	9.4	9.3	0.756
MARD	8.6	8.3	0.101

**Table 4:** Results of post hoc pairwise comparison of the proportion of PDR by cluster

Cluster	SIRD	MOD	MARD
SIDD	0.012*	0.999	0.999
SIRD		0.003*	0.058
MARD			0.999

p-values for Fisher exact test with Bonferroni correction

*Significant results

**Table 5:** Logistic regression analysis of proliferative diabetic retinopathy

	Unadjusted		Adjusted for cluster assignment	
Characteristic	OR (95% CI)^1^	*p*-value	OR (95% CI)^1^	*p*-value
Age (years)	0.98 (0.96 to 1.00)	0.10	1.00 (0.97 to 1.03)	0.85
Sex	0.84 (0.51 to 1.36)	0.48	0.82 (0.49 to 1.34)	0.43
Race/ethnicity				
White	—		—	
Black or African American	0.66 (0.21 to 1.92)	0.45	0.57 (0.18 to 1.69)	0.32
Hispanic or Latino	1.65 (0.82 to 3.61)	0.18	1.40 (0.68 to 3.11)	0.38
Asian	0.54 (0.08 to 2.28)	0.45	0.46 (0.07 to 1.98)	0.35
Other	1.47 (0.30 to 5.80)	0.60	1.28 (0.25 to 5.27)	0.74
Hypertension	0.80 (0.41 to 1.47)	0.49	0.70 (0.36 to 1.31)	0.29
Chronic kidney disease				
No	—		—	
Yes	1.84 (1.10 to 3.07)	**0.020**	1.68 (1.00 to 2.84)	0.051
Uncertain	2.34 (0.91 to 5.59)	0.064	1.98 (0.76 to 4.81)	0.14
Baseline HbA1c (mmol/mol)	1.09 (0.99 to 1.19)	0.078	1.06 (0.93 to 1.20)	0.39
Baseline BMI (kg/m^2)	0.96 (0.92 to 1.00)	**0.039**	0.95 (0.90 to 1.01)	0.093
Age at DM diagnosis (years)	0.96 (0.94 to 0.98)	**< 0.001**	0.969 (0.942 to 0.995)	**0.022**
Baseline microalbumin (mg/dL)	1.004 (1.002 to 1.006)	**< 0.001**	1.003 (1.001 to 1.006)	**0.002**
Albumin creatinine Ratio	1.0003 (1.0002 to 1.0005)	**< 0.001**	1.0003 (1.0001 to 1.0004)	**< 0.001**
Cholesterol (mg/dL)	1.003 (1.0003 to 1.006)	**0.044**	1.00 (1.00 to 1.01)	0.11
Triglycerides (mg/dL)	1.00 (1.00 to 1.00)	0.093	1.00 (1.00 to 1.00)	0.15
HDL (mmol/L)	1.00 (0.98 to 1.02)	0.74	1.01 (0.98 to 1.03)	0.61
LDL (mmol/L)	1.00 (0.99 to 1.00)	0.24	1.0 (0.99 to 1.00)	0.15
HDL/LDL ratio	1.38 (0.67 to 2.63)	0.35	1.69 (0.79 to 3.36)	0.15
ALT (IU/L)	0.99 (0.98 to 1.00)	0.16	0.99 (0.98 to 1.00)	0.21

Bold highlight statistical significance *p* <0.05

^1^*OR* odds ratio, *CI* confidence interval

**Table 6:** Results of post hoc pairwise comparison of the proportion of Vitreous Hemorrhage by cluster

Cluster	SIRD	MOD	MARD
SIDD	0.200	0.999	0.999
SIRD		0.032*	0.005*
MARD			0.999

*p*-values for Fisher exact test with Bonferroni correction

*Significant results

## Discussion

To the best of our knowledge, this is the first study to utilize cluster analysis in evaluating ophthalmic complications in T2DM. Prior cluster studies in diabetes analyzed primarily whether DR was present or not. In contrast, our focus was determining whether certain clusters were associated with DR complications such as PDR, VH, DME, or NVG. Ultimately, being able to determine those that are most at risk of progression or certain complications will allow for targeted prevention and early treatment.

In Ahlqvist and colleagues’ work, they found that SIDD had a significantly higher risk of DR compared with MARD in the ANDIS cohort and that SIDD and MOD had a significantly higher risk of DR than MARD in the ANDIU cohort [1]. Anjana and colleagues studying an Indian population similarly showed that SIDD had the highest risk of DR and that it was significantly higher than MARD [5]. Our analysis showed more specifically that both SIDD and MOD had a higher risk of PDR compared to SIRD. While age at study enrollment did significantly vary across cohorts, SIDD and MOD had the lowest mean ages. This observation was substantiated by the results of logistic regression analysis, which showed that when controlling for cluster assignment, increasing age is associated with a decrease in the odds of PDR. In addition, we found that increases in baseline microalbumin and ACR are associated with an increase in the odds of PDR.

Despite this higher risk of PDR in SIDD, it was the MOD and MARD groups which had significantly higher risk of VH than SIRD. No clear risk factors inherent to the MOD/MARD groups were identified to explain the higher risk VH in these patients. A 2017 study examining levels of VEGF in primary vitrectomy for late VH found significantly higher levels of VEGF in eyes that developed late VH and identified iris neovascularization, hypertension, and proteinuria as risk factors [6]. Hypertension did not significantly vary across clusters in our study to indicate that blood pressure was driving the risk of VH in the MOD and MARD groups.

SIDD appears to have poorer metabolic control as this cluster has the highest average HbA1c on presentation at 12.8 mmol/mol, but also had a significant decrease in HbA1c after establishing care as evidenced by the mean HbA1c of 9.7 mmol/mol over the following 3 years (*p* < 0.001)—a finding which was not observed in the other cohorts. SIDD cluster patients were severely insulin deficient compared to other clusters likely leading to higher elevations of HbA1c when noncompliant compared to other groups that had mild metabolic derangements. The decrease in subsequent HbA1c in these patients was likely due to starting insulin therapy after presenting to medical provider in our system and reflects the importance of initiation of insulin therapy in this insulin deficient group.

While Ahlqvist et al. identified SIRD as having the highest risk of nephropathy, our analysis found that SIRD had the lowest creatinine:microalbumin ratio of the groups and lowest rate of known kidney damage; SIDD meanwhile had the highest. Population differences as well as differences in diabetes duration between the two studies likely contribute to the contradictory findings regarding nephropathy in our study. Given that diabetic kidney disease is uncommon if disease duration is less than one decade [7]. In Ahlqvist’s study, patients were followed for only 10 years after diabetes mellitus diagnosis and could have had different rates of nephropathy compared to our population. Furthermore, there are marked differences in epidemiology of diabetic kidney disease between different ethnic/racial groups with African Americans, Hispanics, and Native Americans having a much higher risk of developing end-stage kidney disease than non-Hispanic Whites [8]. SIRD did have the highest ALT of the clusters in our analysis, which was consistent with both Ahlqvist’s findings regarding SIRD having the highest rate of fatty liver disease and Zaharia’s study demonstrating that SIRD had the highest hepatocellular lipid content at diagnosis and highest rate of hepatic fibrosis at 5-year follow-up [1, 8]. Some of this variability could be explained by different study populations. Our study population was predominantly safety-net hospital population with end stage diseases. Furthermore, significant differences in demographics should be noted between prior studies and our study population.

One of the limitations of our study was that our study population only included patients with DR. Patients without a diagnosis of DR were not included in our cluster analysis as they were not recorded in our database. Therefore, some of the characteristics of our clusters are likely skewed to the severe spectrum of diabetic disease. Associations with other diabetic complications such as nephropathy should therefore be interpreted with caution given the selective cohort. Another limitation of our study was the retrospective nature of our data collection. The longitudinal follow-up of our patients started after diagnosis of DR rather than their initial diabetes mellitus diagnosis. Consequently, some of our patients were either newly diagnosed diabetes mellitus patients or patients with longstanding history of systemic diabetic disease. Also, our study was conducted at a large metropolitan safety-net hospital serving mostly lower socioeconomic patients who were either uninsured or underinsured. Cluster analysis of this population might not be representative of other populations. Furthermore, we observed a relatively large number of subjects with high cluster distance ratio. The distribution of distance ratio in our cohort was similar to what was observed by Kahkoska [2] and may indicate the importance of HOMA2-B and HOMA2-IR (the additional clustering variables used by Ahlqvist) in cluster performance [1].

## Conclusion

Type 2 diabetes mellitus appears to have significant variability in severity, complication risk, and response to treatment. The pathophysiologic cause of this variability is still under study and not yet well understood. A big data approach with cluster analysis of demographic and clinical biomarkers has been used by Alhqvist et al. to help distinguish these phenotypic variants. In our study, we evaluated these clusters for risk of advanced DR and its complications. We found that rates of PDR were higher in MOD, SIDD, and MARD clusters relative to SIRD and that rates of VH were higher in MOD and MARD compared to SIRD. Our study population was selective for patients with DR and thus does not represent the full spectrum of disease of DM, limiting its generalizability to other study populations and potential application to new diabetics. Nevertheless, the diverse population presenting with more advanced stages of diabetes is reflective of many communities within the USA and thus may have certain advantages in terms of generalizability over other international studies. Further research is needed to further define these diabetic variants so physicians can tailor treatment strategies and practice personalized medicine.
